# Simple and automatic monitoring of cancer cell invasion into an epithelial monolayer using label-free holographic microscopy

**DOI:** 10.1038/s41598-022-14034-y

**Published:** 2022-06-16

**Authors:** Ágoston G. Nagy, Inna Székács, Attila Bonyár, Robert Horvath

**Affiliations:** 1 Nanobiosensorics Laboratory, Institute of Technical Physics and Materials Science, Centre for Energy Research, Budapest, Hungary; 2 Department of Electronics Technology, Faculty of Electrical Engineering and Informatics, Budapest University of Technology and Economics, Budapest, Hungary

**Keywords:** Motility, Biological techniques, Biophysics, Cancer, Cell biology, Oncology, Optics and photonics

## Abstract

The invasiveness of cancer cells describes the metastasizing capability of a primary tumor. The straightforward detection and quantification of cancer cell invasion are important to predict the survival rate of a cancer patient and to test how anti-cancer compounds influence cancer progression. Digital holographic microscopy based M4 Holomonitor (HM) is a technique that allows the label-free monitoring of cell morphological and kinetical parameters in real-time. Here, a fully confluent epithelial monolayer derived from the African green monkey kidney (Vero) on a gelatin-coated surface was established, then HeLa cells were seeded on top of the monolayer, and their behavior was monitored for 24 h using HM. Several cancer cells showing invasiveness were detected during this period, while other HeLa cells did not show any signs of aggressivity. It was demonstrated that the invasion of single cancer cells is soundly observable and also quantifiable through monitoring parameters such as phase shift, optical volume, area, and motility, which parameters can easily be obtained and processed automatically. Based on the experimental data, the invasion speed of cancer cells entering the epithelial layer can be defined as the shrinking of detected single-cell volume per unit time. The invasion speed and its correlation with cell migration parameters were analyzed in depth. A clear linear relationship between migration and invasion speed was found, cancer cells with stronger migration have slower invasion speed. These results not only describe the effect of how cancer cells invade the underlying monolayer in contrast to non-invasive HeLa cells, but could help in future research to optimize drugs affecting cell invasibility in a fully automated, label-free and high-throughput manner.

## Introduction

Malignant cancer cells can reproduce themselves without control and penetrate distant tissues via the chain of metastatic events^1^, which is the primary cause of cancer-related deaths^2^. The first step of metastasis is the detachment of individual cancer cells from the primary tumor and invasion into the epithelial cell layer of the host tissue^2–4^. A successful invasion is followed by the intravasation into the circulation and then exiting it with extravasation, after which processes the invading cancer cells can form new colonies in the attacked tissue^3–5^. The dissemination of cancer cells can occur during different stages of tumor progression, and one cellular key-program that initiates this process is the Epithelial-Mesenchymal Transition (EMT)^3–7^. During EMT, epithelial cells receive mesenchymal phenotype, losing the physical connection with the basement membrane, become detached, migrate, degrade the components of the extracellular matrix (ECM) and invade the tissue^4,7^. The transition from collective to single-cell-based migration events can also be accounted for the EMT^6^. Cultured cancer cells (e.g., HeLa) are selected from high-stage cancers that have already become genotypically and phenotypically distinct from the tissue of origin^8^. Therefore, HeLa cells, which are widely used in cancer research and drug development^9,10^, are an ideal candidate to study the metastatic cascade since these cells have already been transformed by EMT and are used worldwide in numerous laboratories^8^. Classic in vitro assays studying cancer cell behavior and drug response include wound healing, gap closure, transwell, microfluidic, 3D platform assays combined with immunofluorescent labeling, and microscopy techniques^11–20^. However, new in vitro*,* label-free, high-throughput techniques are emerging that provide real-time visualization of cancer cell movement, adhesion, division, and pharmaceutical affinity^9,21–27^.

Such an effective, label-free, real-time, and high-throughput instrument is the digital holographic microscopy based M4 Holomonitor^28–30^ (HM). The HM technique uses a diode laser, and a semi-transparent mirror splits its beam to a reference and a sample beam. The sample beam is directed with a mirror through the observed cell culture, and so when the laser light meets translucent objects (e.g., cells and layers) with a specific refractive index, its phase will be shifted compared to the reference light. The reference beam and the sample beam will create interfering wave patterns, which an image sensor camera will then record. Therefore, the primary detected physical quantity is the phase shift of the laser beam caused by cultured cells reconstructing the image of the sample. Since the phase shift of the laser light is affected by the thickness and refractive index of the cells, it is possible to create 3D topographies of the objects in the laser’s path. The constructed images are visualized in the HM software, and parameters related to the kinetics and morphologies of the cells can be exported^31^.

In comparison with other techniques, HM also allows observation in a microfluidic environment, where cells are seeded into a well and chemokines into a different well connected with a microfluidic channel. Samples containing cells are placed on the stage of the HM instrument. The microscope is then focusing on the channel and captures cellular motion in 3D. Holomonitor M4 is an incubator-proof digital holography technique-based instrument^9,32,33^. The main advantage of Holomonitor M4 is the real-time visualization, which does not require the labeling or staining with cytofluorescent molecules, fixation, or any other substance, which could influence the microenvironment of the cells, therefore, it is label-free and cost-effective. The Holomonitor M4 is a precise instrument that monitors cells in a humidified, temperature and gas-controlled environment in real-time, and no post-visualization of the cells is required since the software already calculates cellular features related to dynamics and morphology. These instrumental abilities potentially open up the possibility for the automatic and straightforward detection of cancer cell invasion into compact monolayers, but such investigations were not conducted before.

For this purpose, we used the African green monkey kidney-derived epithelial cells (Vero), which form a closed monolayer^34^ on gelatin-coated surfaces and are ideal for mimicking in vivo processes, for investigating cancer cell invasion, and verifying the invasion events with Holomonitor M4. This cell line has a distinct advantage in methodology development, since the primary endothelial cells are not stable during a long-time culturing and very sensitive to experimental conditions. Using Vero cells was also a choice based on their nature to form compact cellular monolayers, and not their relation to the tissue of origin. The present work describes a phenomenon that occurs when cancerous HeLa cells are seeded on top of the compact Vero monolayer. We demonstrate that holographic microscopy is perfectly suited for 3D monitoring of cancer cell invasion into the underlying monolayer of epithelial cells in vitro. Parameters such as migration, motility, cell area, and volume are measured for invasive and non-invasive cells, and the main discrepancies in the time evolution of these parameters are analyzed. Our study presents a new methodology focusing on the possibility of tracking HeLa cell invasion into epithelial Vero monolayer.

## Materials and methods

### Instrumentation

For the detection of Vero monolayer assembly and HeLa invasion, the digital holographic cytometer Holomonitor M4 (Phase Holographic Imaging PHI AB, Lund, Sweden) was used. During the experiments, the Holomonitor is placed inside a humidified incubator with 37 °C and 5% CO_2_. For imaging purposes, a 35 mm glass bottom dish (VWR) was used, which was covered with a HoloLid (Phase Holographic Imaging PHI AB, Lund, Sweden). The set-up of the Holomonitor and the HoloLid are determined by the manufacturer, and all guidelines were followed according to the manual.

### Cell cultures

Vero (ATCC CCL81) and HeLa (ECACC 93,021,013) cultures were grown and maintained inside a humidified incubator in Dulbecco’s modified Eagle’s medium (DMEM, Gibco), supplemented with 10% heat-inactivated fetal bovine serum (FBS, Biowest), 4 mM L-glutamine, 100 U/ml penicillin and 100 µg/ml streptomycin solution. For Vero cells, 2 mM L-glutamine, sodium pyruvate, and MEM non-essential amino acid solution were specially added into the DMEM. All reagents were purchased from Sigma-Aldrich.

### Measurement protocol

Coating of the 35 mm glass-bottom dish was performed by placing the dish inside the incubator at 37 °C with 1 ml 0.5% gelatin-PBS solution for 20 min. After the incubation, the coated dish was rinsed three times with PBS and was filled with 3 ml complete cell culture media until further use. The ultra-thin gelatin layer applied was already characterized previously by our group with QCM, OWLS, and Epic BT label-free biosensors and atomic force-microscope (AFM), and its thickness was measured to be around 16 nm^35^.

**Figure 1:**
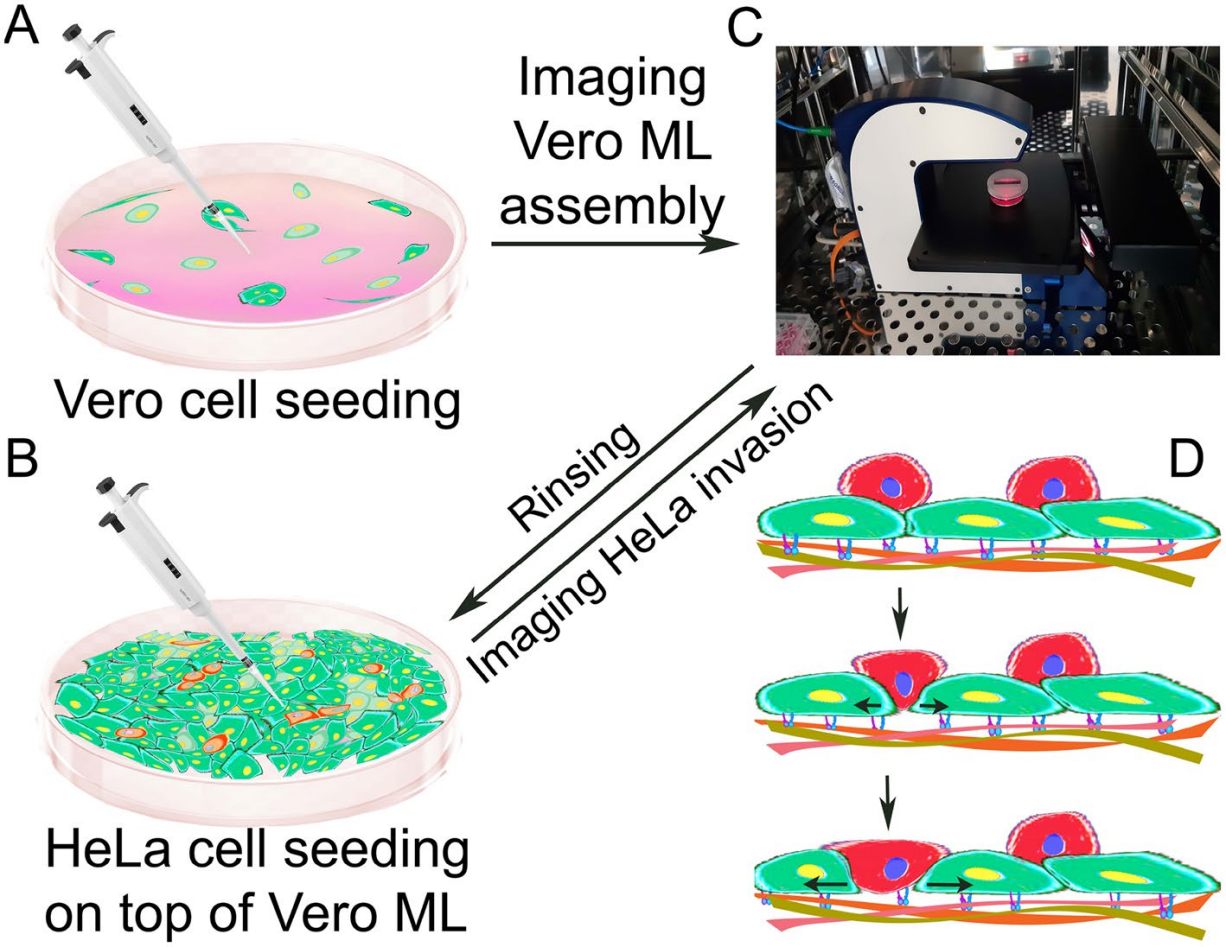
Schematics of experimental design, execution, and results. (**A**) Seeding of Vero cells (green) on gelatin-coated dish. (**B**) Seeding of HeLa cells (red) on top of the self-assembled 100% confluent Vero monolayer (ML). (**C**) Holomonitor M4 was used to image monolayer assembly and invasion for 24 h. (**D**) Illustration of the expected result, the HeLa cells seeded on top of the Vero monolayer infiltrate by searching for optimal invasion positions.

**Figure 2:**
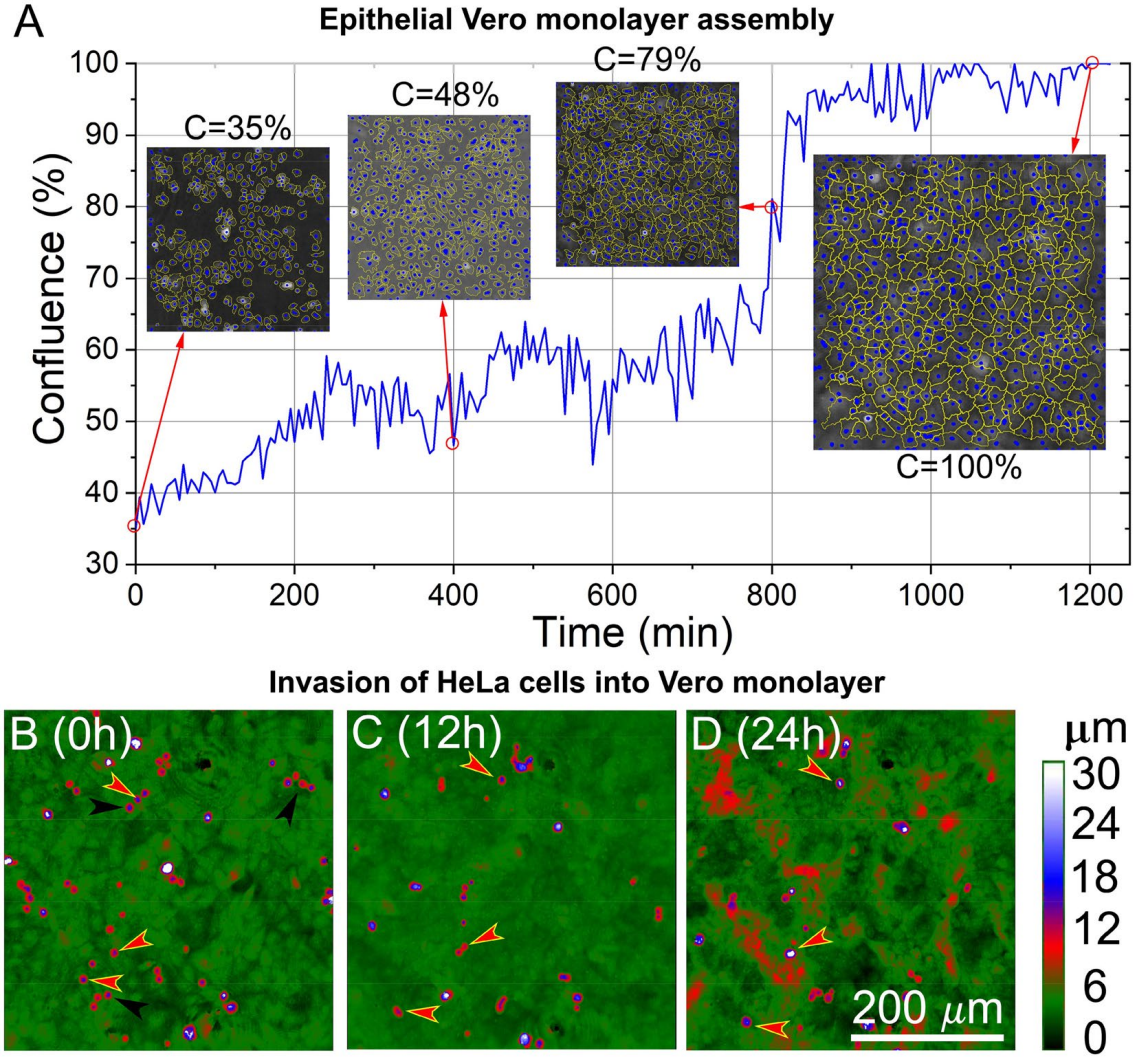
Maturation of the epithelial Vero monolayer and the invasion of HeLa cells. The eptihelial Vero monolayer was 100% confluent after 1200 min observed and quantified with HM (**A**). (**B**–**D**) Seeding, migration, and invasion of HeLa cells (0–12–24 h); It is visible that after 12 h of recording (**C**), some cells diminish from the view because of invasion and migration outside the frame. After 24 h of seeding HeLa, the smooth Vero monolayer (**D**) became rough and textured. Black arrows mark the three invasive, and red arrows with yellow outline mark the three non-invasive HeLa cells selected for detailed analysis.

Vero cells were picked up from confluent sustained cell cultures by rinsing the cells with DPBS followed by adding 0.05% (w/v) trypsin- and 0.02% (w/v) EDTA-PBS solution for 2 min to detach cells from the tissue culture dish. Detached cells were washed with 1 ml of completed medium, and 200 µl cell suspension (~ 6 × 10^5^ cells) were added to the 35 mm 0.5% gelatin-coated dish. The gelatin-coated dish was covered with the HoloLid, which assembly was placed on the stage of the M4 Holomonitor located in the humidified incubator (Fig. 1). During 24 h of monitoring, the assembly of a 100% confluent Vero epithelial monolayer could be observed (Fig. 2.).

HeLa cells were also picked up from a confluent sustained cell culture with the same procedure as Vero cells. To visualize invasion, after rinsing the confluent Vero monolayer with completed cell culture media and refilling the dish, 100 µl HeLa cell suspension (~ 1.2 × 10^5^ cells) was added on top of the fully confluent Vero monolayer (Fig. 1), for which step the recording on the Holomonitor M4 was paused and the HoloLid covered dish was removed from the stage. After the injection of HeLa cells onto the monolayer, the dish was covered again and placed back to the stage of the instrument, and recording was immediately continued. The recording was proceeded for 24 h to determine the invasive nature of HeLa cells (Fig. 2).

### Data acquisition and evaluation

The software HStudio M4 (Phase Holographic Imaging PHI AB, Lund, Sweden) is used by the Holomonitor M4 instrument to visualize, detect, track and determine cell morphology and movement. HStudio can export obtained data for further processing and data analysis, which was done in OriginPro 9.5.

**Figure 3:**
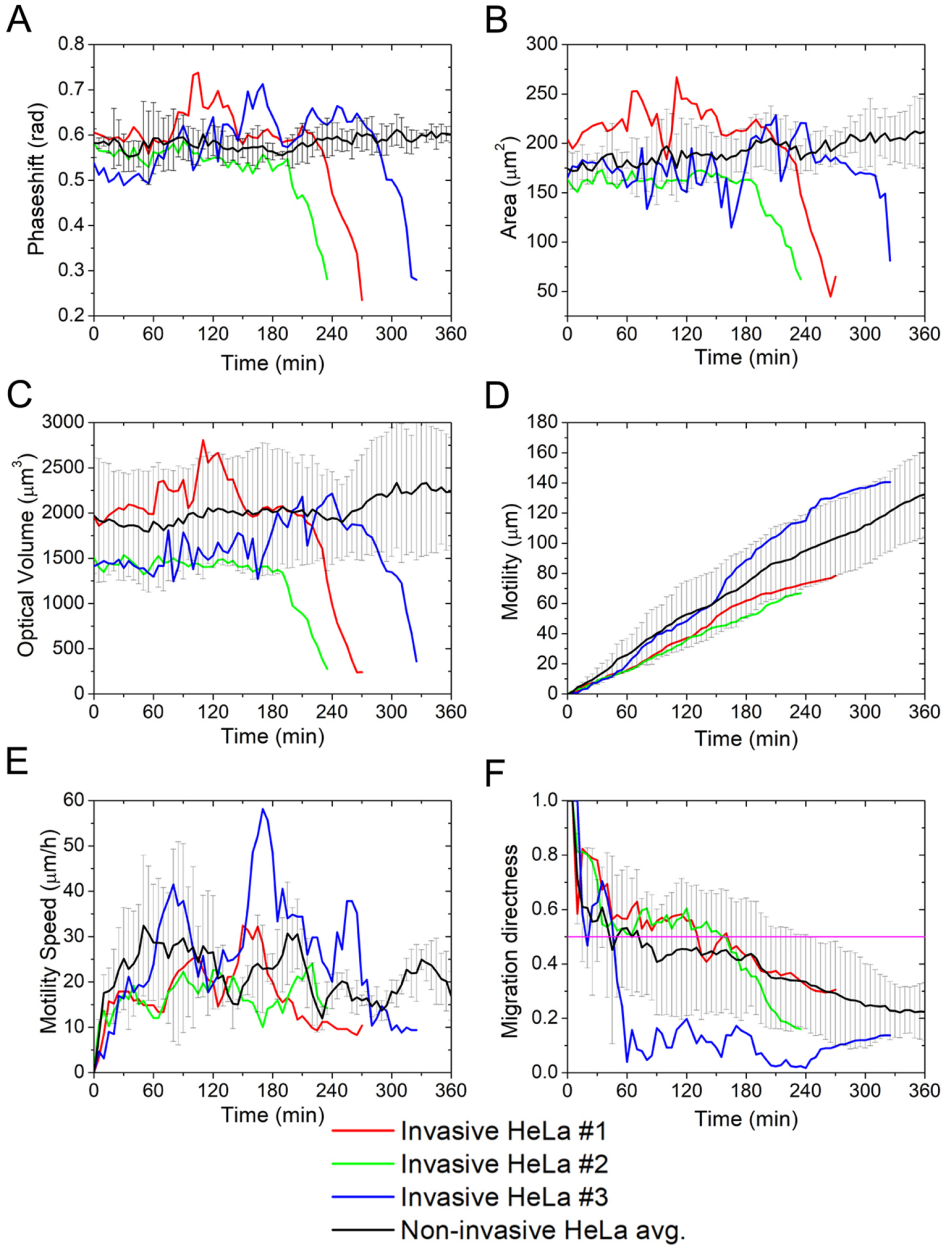
Phase shift (**A**), area (**B**), optical volume (**C**), motility (**D**), motility speed (**E**) and migration directness (**F**) of individual invasive (red, green, blue) and non-invasive HeLa cells. The black curve marks the average with the error marked with grey drop lines.

The acquired data were used to plot the motility and morphology of the selected three invasive and three non-invasive HeLa cells (Figs. 2, 3). This selection was based on the same parameters, such as the time frame of invasion, the area, and the optical volume of the cells, since it is important to have almost the same characteristics when comparing the invading and non-invading type of HeLa.

## Results

The investigations have shown that during the experiments, some of the seeded HeLa cells on the top of the confluent monolayer invade, while some other HeLa cells are non-invasive and remain (and only migrate or divide) on the top of the monolayer, during the observed time period (Supplementary Information (SI) movie_HeLa-Invasion_All). We also address the confluency of the epithelial Vero monolayer quantitatively, and Fig. 2 shows the establishment of the monolayer in real-time. Basically, full confluency is reached in 1200 min (20 h), and HeLa cancer cells are always added after reaching this state. In total, 20 HeLa cells seeded on top of the Vero monolayer were chosen for evaluation from a 24-h window. The *n* = 20 investigated HeLa cells contained *n* = 12 invasive and *n* = 8 non-invasive HeLa cells, and their parameters such as area, optical volume, motility, migration, motility speed, and migration directness were obtained. From the 20 HeLa cells, three invasive and three non-invasive HeLa cells were selected for demonstrative purposes (Fig. 2 and SI movie_HeLa-Invasion1-2-3). (Note, experiments with more HeLa cells added were also performed, showing similar behaviours. (see SI movie_HeLa_invasion_All2.avi) These 3–3 cells had similar morphological features, however, based on their behavior, cells could be distinguished as invasive and non-invasive types. Invasion is characterized by a loss in the signal, which means that the software can not track the desired cell anymore since it is indistinguishable from the underlying monolayer. Therefore a simple observation could be made, namely, non-invasive cells stay on top of the monolayer by being visible since they do not merge, while invasive cells find a spot to infiltrate the monolayer, in the observed time frame. An additional control experiment with HeLa cells was also performed without Vero cells using the same gelatin coating protocol (see SI movie_HeLa-on-gelatin.avi). During the 24-h recording, migration and mitosis of cancer cells could be well observed, but no invasion type signal was seen on the gelatin layer. The results are as expected due to the previously measured thickness of the gelatin coating (16 nm)^35^.

The results show that the phase shift, area, and optical volume contain a drop in the signal that is characteristic of invasive HeLa cells (Fig. 3). Motility, describing the total path traveled by a cell, on average, is linearly rising, even when single invasive HeLa cells are compared to the average of non-invasive HeLa cells. However, the loss in the signal is important in the sense of data analysis since it marks the final spot and time of the invading cell. Importantly, invasive and non-invasive HeLa cells cannot be distinguished based on parameters such as migration speed and migration directness. Also, migration directness is proof that cells were allowed to migrate freely on the Vero monolayer since its value is below 0.5 (Fig. 3).

**Figure 4:**
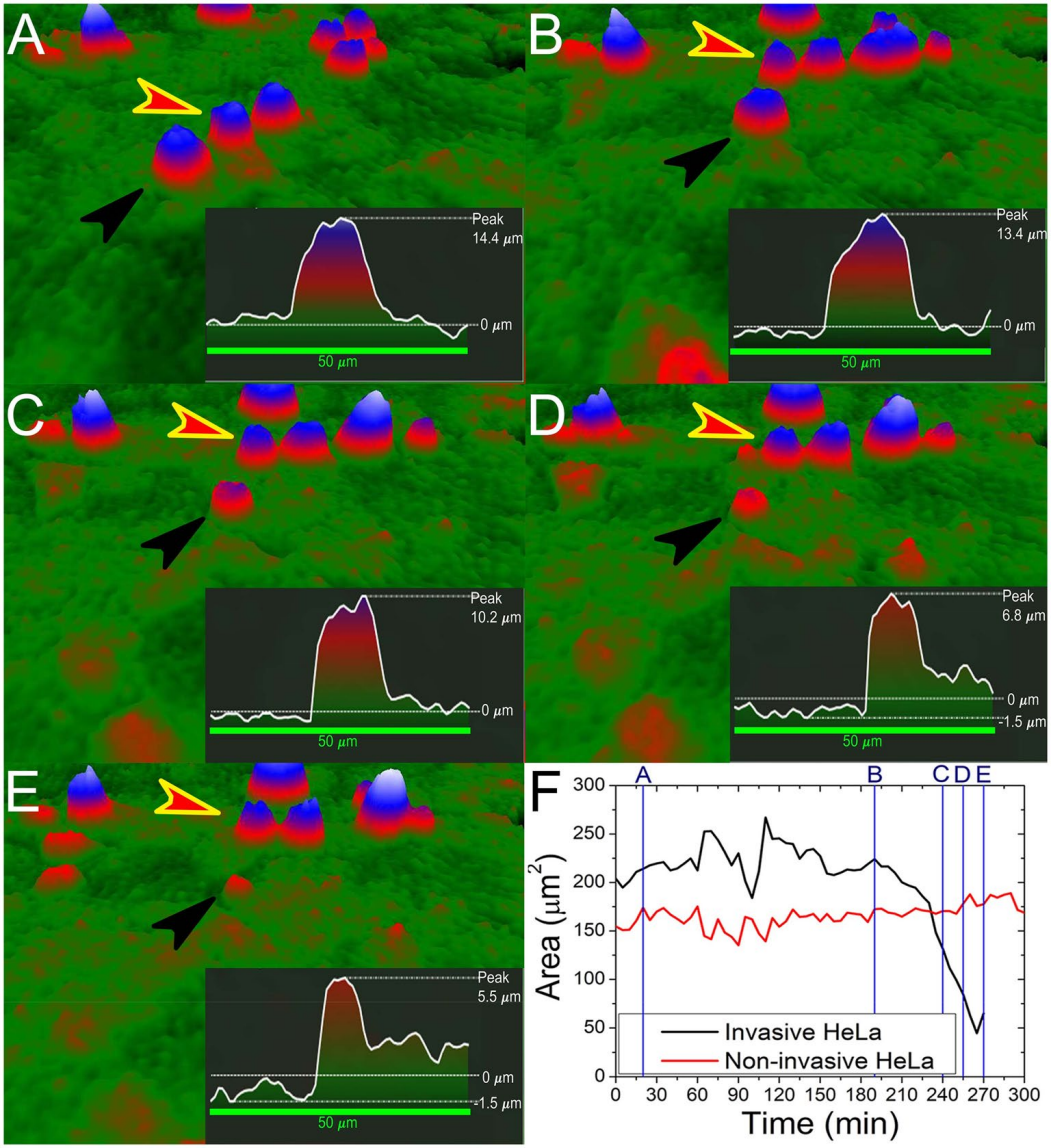
Comparison of invasive and non-invasive HeLa cells. (**A**–**E**) Black arrowheads indicate the invading, while red arrowheads with yellow outlines indicate the non-invading HeLa cells. A 50 µm length cross-section of the invading cell is present in each picture. Cross-sections show a decrease of the peak value, while the average 0 level is rising, which is the consequence of the infiltration of the cells into the gap. (**F**) Graph of the area over elapsed time since seeding. The drop in the parameter is an indicator of invasion blue lines correspond to the 3D pictures in time to (**A**–**E**) displaying stages of infiltration: (**A**) 20 min, (**B**) 190 min, (**C**) 240 min, (**D**) 255 min, and (**E**) 270 min.

**Figure 5:**
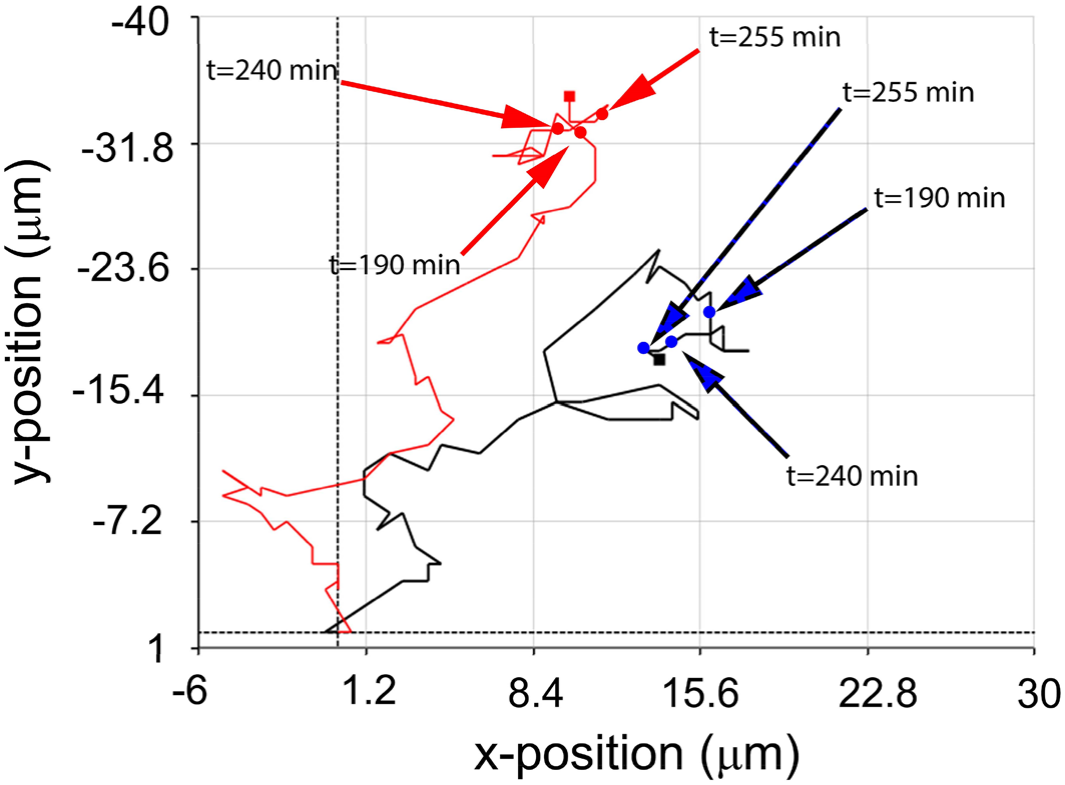
Tracking and movement of the invasive (black) and non-invasive (red) HeLa cells presented in Fig. 4. Each graph presents the cells’ position at a specified time elapsed since seeding: 190 min, 240 min, 255 min, and 270 min. The invasive cell circles around a possible spot optimal for infiltration of the underlying Vero monolayer.

**Figure 6:**
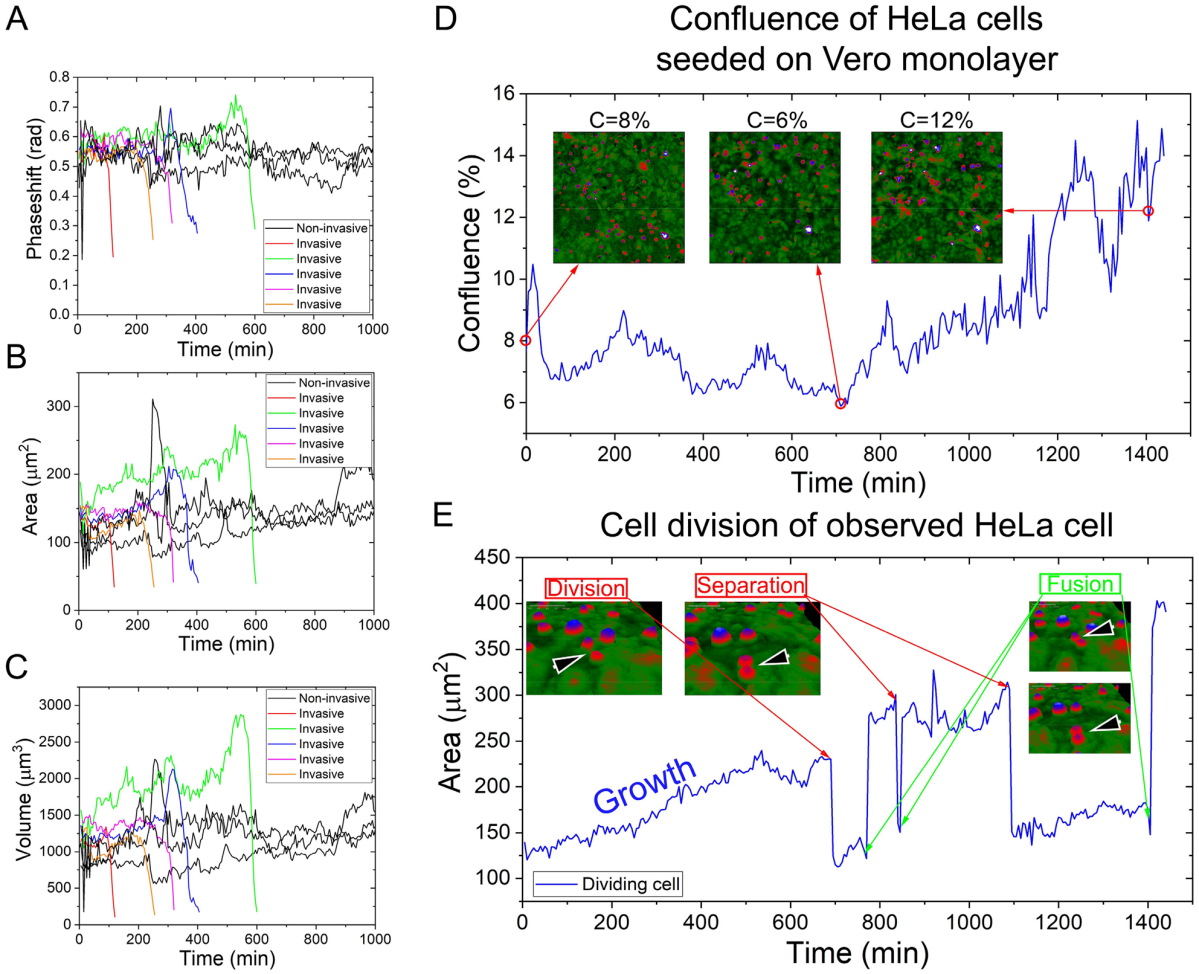
Results from repeated experiments show the same tendency in the phaseshift (**A**), and morphology parameters area (**B**) and volume (**C**). The HeLa cells seeded on top of the monolayer have invasive and non-invasive profiles, and the non-invasive cells divide after a certain amount of time predictable by the timespan of the cell cycle, which is visible in the confluence of the seeded HeLa cells on the monolayer over time (**D**). Non-invasive dividing HeLa cancer cells have special morphology and movement characteristics, since after division the daugther cells do not invade the epithelial Vero monolayer, but remain on top and fuse then separate multiple times observable by morphology parameters such as area (**E**).

A detailed analysis of adjacent invading and non-invading HeLa cells revealed that during the same time period, the non-invasive HeLa cell’s area remains the same, while the invasive HeLa has a drop in the observed area (Fig. 4). It must be emphasized that the area and optical volume are the only parameters analyzed here that are calculated by the evaluation software based on the phase shift of the laser beam. The actual volume and area of the cell can be considered constant. Although there is a fluctuation in the measured signal, fitting a linear on the phase before invasions shows that the area and volume do not change much in this period (Fig. 3). Thus a sudden drop in the measured area/volume can be directly associated with the invasion of these cells. The tracking curve describes the migration of the cells on the XY plane, and it shows how an invasive cancer cell searches for a spot optimal for invasion processes, which is seen as a circling of the invasive cell around a possible spot (Fig. 5). We observed that when cells reach the invasion spot, the migration is narrowed down to a few micrometers (Fig. 6). Importantly, cross-section of the invading cell revealed that the invading HeLa cell has the capacity of filling up gaps. On the tracking, it is visible that the invading HeLa cell circles around a spot, which seems to be an ideal part of the underlying confluent monolayer to carry out the invasion. This position can be considered as some sort of gap (as seen in Fig. 4) when interpreting the cross-section of the HeLa cells and their vicinity. A 50 µm cross-section of the cell was taken, which revealed a slight difference between the levels of the neighboring cells. The gap can be seen on the right side of the cross-section compared to the left side, which is taken as level 0 (Fig. 4). This gap is gradually filled up and elevated during the invasion, meaning that the invading cell infiltrates the layer, causing a rougher texture. The experiments were repeated several times, with both cell lines undergoing a minimum of 2–3 passages, and the primary behavior of the cells, especially the presented holographic microscope signals characterizing cancer cell invasion, were well reproducible. In order to highlight this, we present more data and relevant analysis in Fig. 6A–C. The invasion of HeLa cancer cells into the monolayer can also be monitored when addressing the confluency of the seeded cells. In the beginning, a certain number of cells are in the visual area of the Holomonitor, which value starts to drop immediately and consistently until a certain time point, as shown in Fig. 6D. However, since non-invasive cells remain on the top of the monolayer, the confluency starts to rise due to the cell divisions occurring above the monolayer. The signals of the instruments can characterize the mitotic events well. Interestingly, daughter cells of the dividing HeLa cells tend to remain close to each other and fuse, then separate again as they continue to migrate on top of the monolayer shown in Fig. 6E.

A further parameter we introduced here is the speed of invasion ${(v}_{invasion})$, quantifying the speed of detected single-cell volume shrinkage in time:

1 $${v}_{invasion}= \left|\frac{{\Delta V}_{cell}}{\Delta t}\right|$$

**Figure 7:**
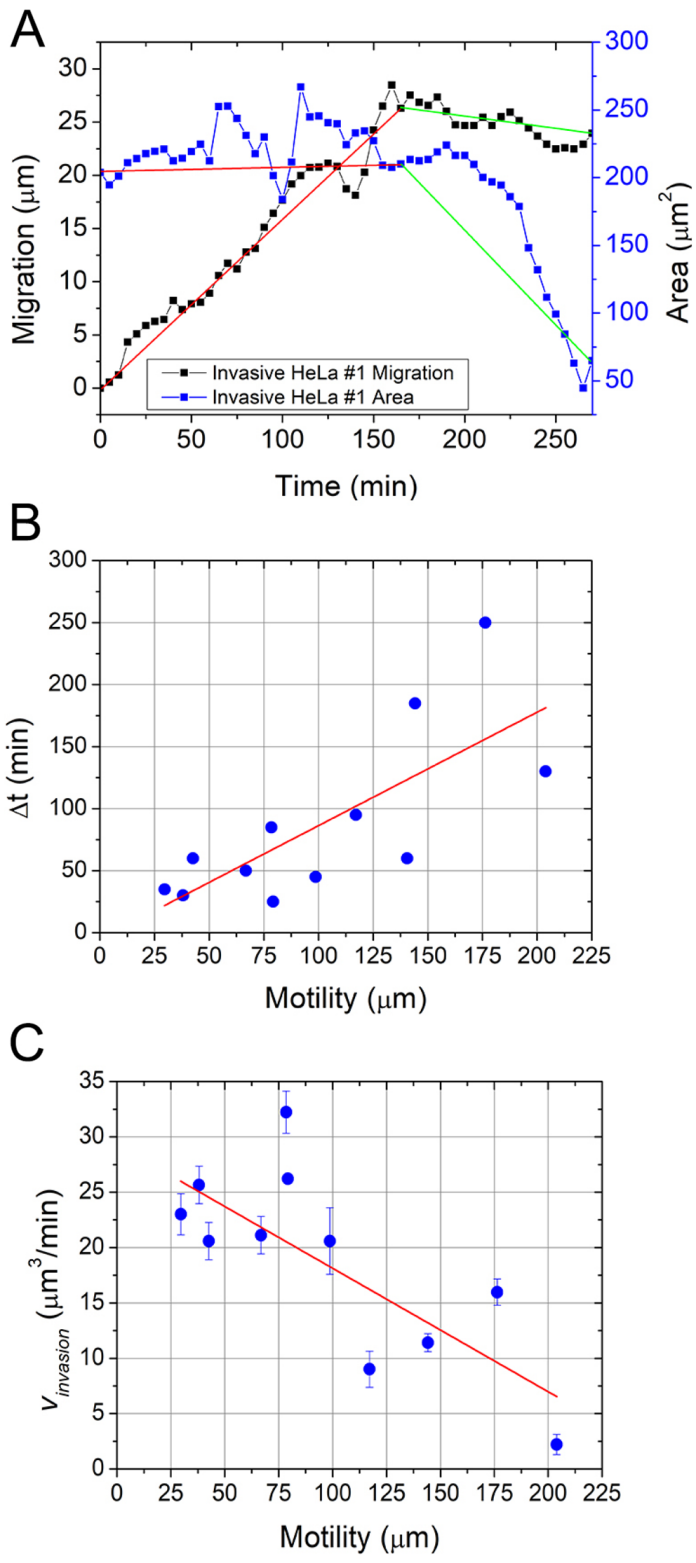
(**A**) The example shows invasive HeLa cell #1 during the movement phase (red, 0 < ${t}_{m}$<165 min) and in the lock-in phase (green, 165 < ${t}_{l}$<270). (**B**) Elapsed invasion time (∆*y*) over motility shows a correlation of *r* = 0.743. (**C**) Derivate slope ${v}_{invasion}$ correlates (*r* = -0.768) with motility.

The evaluation is based on the separation of the processes into stages before and after the drop in the measured signal of phase shift, area, and volume. The elapsed time (∆*t*) is the time necessary for the HeLa cell to infiltrate into the underlying Vero monolayer. Since morphological parameters vary among cells, it is adequate to treat their signals according to their sizes. The origin of the elapsed drop time (∆*t*) is the commence of the drop in the signal. However, for the determination of ${v}_{invasion}$ a decrease by 30% of the average value in a morphological parameter was used (e.g. ${\Delta V}_{cell}$). Interestingly motility, which parameter describes the total path traveled by a cell, shows a correlation with the speed of invasion. It can be observed that the further a cell traveled, the smaller ${v}_{invasion}$ is (see Fig. 7).

Moreover, an interesting phenomenon could be observed related to the migratory behavior of the HeLa cells. The data and the plotted graphs suggest that two distinct phases exist in the movement of the cancer cells on the monolayer during migration. The two phases, movement phase $({t}_{m}$), and lock-in phase $({t}_{l}$), are particularly visible in the invading type of HeLa cells. Invading HeLa cells show a reduction in morphological parameters phase shift, area, and optical volume, which drop in the signal is synchronous with the lock-in phase of the cells, in contrast with the movement phase where the mentioned parameters are stable, and the variation is small. We can assume that during the movement phase, the cancer cell searches for a possible spot for invasion in the underlying monolayer. The change (*Q*) in the movement phase and the lock-in phase can be quantified by dividing the cell area (${\Delta A}_{cell}$) with the total displacement ($\Delta r)$ of the cell during an observed phase (${t}_{phase}$) (Eq. 2). The movement phase has, therefore, a lower *Q* value compared to the lock-in phase where the invading cancer cell stays around a self-defined position and invades the monolayer resulting in the decrease of morphological parameter values and, finally, the loss of its signal (Fig. 7).

2 $$Q= \left|\frac{{\Delta A}_{cell}({t}_{phase})}{\Delta r({t}_{phase})}\right|$$

The example in Fig. 7 shows invasive HeLa cell #1 during the movement phase (red, 0 < *t* < 165 min) ${\Delta A}_{cell}$(${t}_{m}$) = 82.97 µm^2^ and $\Delta r({t}_{m})$=28.48 µm yielding *Q* = 2.91 µm, however, in the lock-in phase (green, 165 < *t* < 270) ${\Delta A}_{cell}$(${t}_{l}$) = −179.4123 µm^2^ and $\Delta r({t}_{l})$ = 5.05 µm yield *Q* = 35.53 µm. This type of evaluation shows that the invasion of a cell is quantifiable, and a relation between the movement, the area, and the invasion of HeLa cells exist.

## Discussion

The investigation of the invasive nature of HeLa cells on a confluent monolayer of epithelial Vero cells has proved to be a valuable model for future research and development, and could demonstrate the most important features present during cellular invasion processes. It was shown that it is possible to determine and quantify invasion with the digital holography technique using Holomonitor M4. Factors on which an invasion can be quantified are cell morphology features such as area and volume, and behavioral parameters, including motility, invasion speed, and change in the movement and lock-in phases of the cell. These parameters can be used for the automatic detection of cancer cell invasion into monolayers under environmentally controlled conditions.

We found that the motility of invasive HeLa cells falls within the average and error range of non-invasive HeLa cells. The discovered loss in the holomonitor signal due to cancer cell invasion could greatly help in the development of automated detection methods in the future. For example, when investigating large samples, the linearly rising motility curve would be broken at some point due to the signal loss and identification of invasive cancer cells could be done based on this unique feature. Moreover, when using the evaluation software of Holomonitor M4 the loss of signal can also be considered as easily detectable in all parameters because the software tries to continue the tracking even when the original cell has already vanished from the sight. Therefore, based on our results, a sophisticated method could identify automatically if invasive processes were present or not, and could supply the ratio of invasive cells in a population at a given biological setting. Invasion speed and the change in phases are calculated parameters that are unique to all cells, but a correlation of invasion speed and motility could be first observed in our experiments. This type of quantification of cellular behavior could be used in the future for invasion detection of cancer cells. In short, the longer a cell migrates on the monolayer, the flatter its infiltration curve is and therefore has a lower invasion speed. The presented methods can be valuable to determine cancer aggressiveness and the effect of anti-cancer drugs on the cell invasion process.

Studies have found that cancer cell invasion is possible on the single-cell and also on a collective level^36^, and here we have shown a single-cell case study, where individual cancer cells invade the underlying epithelium. The invasion of single cells may remodel the ECM^37^, therefore the use of matrix-metalloprotease (MMP) inhibitors would enable to study if HeLa and other cancer cells can invade the monolayer regardless of secreted MMP-s^6^. Also, MMP-enhancer substances (e.g., pro-MMP-s) would show if cancer cell invasion capability is a product of MMP secretion and all of the cells in the view of the sensor could invade, or there may be some phenotypical plasticity that enabled a proportion of the studied cells to invade and another proportion to stay on top of the monolayer. It is important to mention that our investigations involved two types of cells, namely Vero and HeLa, so future investigations with the proposed technique give the possibility to include other cancer cell lines to have more data on their invasive nature to enable the investigations of primary tumors with HM imaging and study the effect of anti-cancer^9^ and-metastasis drugs. Based on the literature, surface proteins VCAM, PECAM, ICAM, EPCR, and TM interact with integrins, which connections are responsible for intercellular adhesion between the endothelium and white blood cells and cancer cells^38,39^. Accoring to our observation, there is an adhesion promoting and maintaining cell–cell interaction between Vero and HeLa cells, promoting and maintaining adherence of cancerous HeLa cells accounting for the observed invasion characteristics^40,41^.The in vitro technique presented by us is only mimicking a fraction of the whole in vivo processes, so it would be highly desirable to refine this experimental set-up. Placing endothelial cells instead of epithelial cells on the surface, and adding blood-related elements and substances to the culturing media would enable to study the behavior of complex systems. For example, the addition of macrophages to the serum substitute would show their role in the intra-or extravasation of cancer cells through an endothelial barrier^42,43^. Also, in vivo cancer cells can modulate their invasion characteristics over time, suggesting a certain plasticity during cancer cell invasion^36^, therefore our proposed method is able to simply distinguish between such events (e.g., single-cell invasion vs. collective invasion) in vitro compared to e.g., 3D ECM invasion assays, because of the real-time and label-free acquisition method of the HM instrument. The instrument is used for many applications, including substance screening, cellular behavior, wound healing assays and cell sheet applications^44,45^. The applicability of ECM mimicking gels was previously verified by Hellesvik et al.^46^, and in our application, the thickness of the gelatin layer inhibited the migration of cells through the gel. The approach presented in our manuscript is mainly a technical demonstration that the Holomonitor M4 can be used to effectively track and measure cancer cell invasion into cell sheets in a simple label-free manner.

Previous works mainly employed fluorescent microscopy and labeled cells to measure the invasion process in ECM mimicking gels (Collagen^15,16,19^, Matrigel^18^). This requires additional reagents and the employed fluorophores can affect the physiological state of the cells. Table 1. summarizes the previously published methods available for cancer invasion studies and shows the main advantages and disadvantages of the techniques. Clearly, the proposed methodology in the present work is unique in several aspects. First, labeling is not needed, cells can move freely on the surface, and their migration is not limited to a certain direction. Second, the cancer cells are invading a tight epithelial monolayer, which in vitro methodology resembles the in vivo process of invasion. Third, invasion of cancer cells is quantifiable, and invasion parameters can be extracted in an automatic manner. Moreover, the scaling up of the investigations is straightforward by employing motorized stages and multiple wells on the HM instrument.

**Table 1:** Collection of previously published methods and results from cancer cell invasion studies.

Method	Cell lines	Results	Advantages	Disadvantages	References
Videorecording and cell tracking	Primary tumor cells: oral-, breast- and rhabdomyosarcoma. Control: normal mucosa	Primary tumor cells migration occurred after 3–12 days from seeding in a 3D collagen matrix model, migration speeds were recorded up to ca. 15–70 µm/h. Cell clusters showed directional movement, while single-cells exhibited random migration patterns	Simple methodology; Cost effective; Label-free	Extraction of morphological parameters is not possible. Low-throughput	^13^
Three-layered 3D microfluidic platform	MDA-MB-231 and MCF-7 breast cancer cell lines, HUVEC endothelial cells	The novel platform identified increased invading capability of MDA-MB-231 cells compared to MCF-7 cells into HUVEC cell layer. MBD-MB-231 cells decreased permeability and diameter of the vasculature	Multipurpose use with the ability to introduce flow; 3D	Low-throughput. Requires a special microfluidic cell; Not label-free; No morphological or motility parameters extracted	^14^
MI-Chip; Fluorescent microscopy	SUM-a59 and MCF-7 breast cancer cell lines	Cancer cells with different densities seeded into the MI-Chip microwells showed migration towards cues placed on top of the collagen gel containing wells. The technique is opening up possibilities to study 3D cancer cell invasion in a high-throughput manner	High-throuput; 3D; real-time	Not label-free: cells are labelled with GFP; No morphological or motility parameters extracted	^15^
Motorized stage microscope	HT-1080 fibrosarcoma, LNCaP, Du145, PC3 prostate cancers, MBD-MB-231 breast cancer cell lines	The study included the invasion of multiple cancer cell lines in 3D spheroid structures into collagen gel under standard conditions and in the presence of drugs	High-throughput; 3D; real-time; simple technique	Not label-free; No morphological or motility parameters extracted	^16^
Transwell assay	Hepatocellular carcinoma cell lines and primary tumor cells	Small nucleolar RNA host gene 20 is responsible for cell proliferation, invasion and the expression of certain genes	Simple technique; cancer cells penetrate cellular monolayer	Not label-free; Results only show the presence of migration and the number of invading cells	^17^
3D-ECM Transmigration and invasion assay	MBA-MD-231 breast cancer cell line, HUVEC, HPMEC and HDMEC endothelial cells	Cancer cells seeded on top of the endothelial monolayer showed migration towards the 3D ECM gel. Transmigration was inhibited by knock-down of the integrin α5 subunit. Decreased stiffness and cytoskeletal remodeling due to small GTPase activation led to an increased invasion	Simple technique; cancer cells penetrate cellular monolayer	Low-throughput; Not label-free; Results only show the presence of migration and the number of invading cells	^19^
Wound closure assay, Spheroid basement membrane invasion assay, proliferation assay	HT29 and HCT-116 human colon cancer cells,	Aquaporin-1 inhibition reduced invasion capabilities of HT29 cells, but did not influence HCT-116 cells. Inhibition reduced tube formation in endothelial cells	Simple techniques	Low-troughput; 2D; Not label-free	^18^
Resonant waveguide grating (RWG) Biosensor	HT-29 colorectal adenorcarcinoma cell line	Invasion of spheroidal cancer cells into 3D Matrigel structure above RWG sensor surface. Investigation of drugs is possible	High-troughput; Real-time; Label-free; 3D	Special knowledge on optical biosensors is required; expensive set-up and RWG plate. Invasion measurement through cellular monolayer needs further development	^22^
Digital Holography	HeLa cervix carcinoma cell line and Vero kidney derived epithelial cell line	Invasion of multiple cancer cells into cellular monolayer was observed. Characteristic invasion curves were distinguishable from non-invasive cells	High-throughput; Real-time; Label-free; Measurements are cost-effective and simple; Incubator proof; Automatization of invasion detection and quantification; 3D; cancer cells penetrate cellular monolayer	Requires a special set-up	Present work

HM enables a real-time and label-free approach, which is optimal for the detection of invasion of cancerous HeLa cells into a confluent Vero monolayer grown on gelatin.

Summarized, the results show a new opportunity to study cancer cell invasion into cellular monolayers in depth. Most of the prior studies (Table 1) investigated the relative number of migrating cells into gels, since they did not investigate motility or morphology parameters further^14–19^, due to the fact that most of the methods are not capable of extracting real-time motility and morphological parameters, compared to the parameters calculated by the software of the Holomonitor M4^47^. However, investigations on the movement speed of cancer cells in gels have similar results to ours^13^. Also, methods capable of monitoring the speed of invasion in an incubator-proof and label-free manner are relatively rare^22^. The Holomonitor system can be extended with high-throughput applications with a motorized xy-stage that allows monitoring of up to 96-wells. Also, combination of the instrument with fluorescent mode could capture fluorescent images in real-time with short pulses of laser illumination, which would presumably not interfere with a cell’s normal homeostasis. With the help of holomonitor also the establishment of 3D spheroids could be monitored^48^. However, some drawbacks can only be eliminated by the aforementioned combined techniques, since Holomonitor does not allow specific, or molecular level single-cell analysis. Also, the instrument is incubator proof and so requires a high-precision maintenance and handling. HM has the potential for automatic detection of cancer cell penetration of confluent monolayers, the possibility to extend the measurements to multiple wells and cell types gives the ability to test new substances and analyze the motility and morphological parameters of invading cells in a high-throughput manner.

## Supplementary Information

Supplementary Information 1.

Supplementary Video 1.

Supplementary Video 2.

Supplementary Video 3.

Supplementary Video 4.

Supplementary Video 5.

Supplementary Video 6.

## Data Availability

All data generated or analysed during this study are included in this published article and its supplementary information files.
