# Supplementary material for: Simple and automatic monitoring of cancer cell invasion into an epithelial monolayer using label-free holographic microscopy
Source: Sci Rep. 2022 Jun 16;12:10111. doi: 10.1038/s41598-022-14034-y (PMC9203807; doi:10.1038/s41598-022-14034-y)
Supplement: Supplementary file 1 — Supplementary Information 1. [file 41598_2022_14034_MOESM1_ESM.docx]

Supplementary Information

for

Simple and automatic monitoring of cancer cell invasion into epithelial monolayers using label-free holographic microscopy

Ágoston Gábor Nagy^1,2)^, Inna Székács^1)^, Attila Bonyár^2)^ and Robert Horvath^1)^

1) Nanobiosensorics Momentum Group, Institute of Technical Physics and Materials Science, Centre for Energy Research, Budapest, Hungary.

2) Department of Electronics Technology, Faculty of Electrical Engineering and Informatics, Budapest University of Technology and Economics, Budapest, Hungary

**movie_HeLa-Invasion_All.avi** Recording of the HeLa movement of the confluently assembled Vero monolayer shows invasive, and also non-invasive cancer cells (which remain on top of the monolayer throughout the whole recording time (24 hours)). The confluency of Vero monolayer was also tested and full confluency was reached in 1200 minuted (20 hours) after seeding the Vero cells on the gelatin substrate.

**movie_HeLa-Invasion1-2-3.avi** The movies show invasive HeLa cancer cells selected for demonstration purposes on top of Vero monolayer.

**movie_HeLa-Invasion_All2.avi** Control experiments by adding more HeLa cells on top of the monolayer.

**movie_HeLa-on-gelatin.avi** An additional control experiment was carried out by only adding HeLa cells to the ultra-thin gelatin layer, which study did not find any invasion like signals of the cancer cells. (The layer and the cell seeding was prepared with the same coating and the same seeding protocol as previously.)
